# Purchase of food for away-from-home consumption according to urban and rural areas of Brazil between 2002 and 2018

**DOI:** 10.1590/1980-549720250013

**Published:** 2025-04-07

**Authors:** Thais Meirelles de Vasconcelos, Anna Karolyne Pontes de França, Marina Campos Araújo, Ilana Nogueira Bezerra

**Affiliations:** I Universidade Estadual do Ceará, Graduate Program in Public Health - Fortaleza (CE), Brazil.; II Universidade Estadual do Ceará, Health Sciences Center - Fortaleza (CE), Brazil.; III Escola Nacional de Saúde Pública Sergio Arouca, Graduate Program in Public Health Epidemiology - Manguinhos (RJ), Brazil.; IV Universidade Estadual do Ceará, Graduate Program in Nutrition and Health - Fortaleza (CE), Brazil.

**Keywords:** Feeding Behavior, Eating, Food surveys

## Abstract

**Objective:**

To analyze the differences in the evolution of purchasing food for consumption away from home between urban and rural areas of Brazil, from 2002 to 2018.

**Methods:**

Population data from 245,711 adults (25-59 years old) from the Consumer Expenditure Survey, of the Brazilian Institute of Geography and Statistics, of 2002-2003, 2008-2009, and 2017-2018 were used. The foods were grouped into ten nutritional and marketing categories and the frequency of purchasing food for consumption outside the home was compared according to urban and rural areas of Brazil and geographic regions of the country. Differences between areas were considered when the 95% confidence intervals of the estimates did not overlap.

**Results:**

We found an increase in the purchase of food for consumption outside the home in urban areas between 2002-2003 and 2008-2009, followed by a reduction in 2017-2018. In rural areas, the frequency remained stable over the years. Regarding the food groups, there was a reduction in the purchase of soft drinks and alcoholic beverages and an increase in the frequency of meals away from home. The differences between urban and rural areas varied according to the regions of Brazil.

**Conclusion:**

The purchase of food for consumption outside the home between 2002 and 2018 was different in urban and rural areas of the country; nevertheless, the rural area is approaching the urban area. These results highlight the new context of rural life and the need to evaluate eating behaviors in this area.

## INTRODUCTION

The increased participation of eating away from home in the diet and its negative health impacts are recognized in developed and developing countries^1^ ^,^ ^2^. Some explanations focus on demographic and socioeconomic changes that have modified eating behaviors^3^. In addition, consumption outside the home is related to the intake of high calorie density foods, greater consumption of foods high in saturated fat, and lower fiber intake^4^ ^,^ ^5^, contributing to the development of Chronic Noncommunicable Diseases (NCDs)^6^.

According to the latest Consumer Expenditure Surveys (*Pesquisas de Orçamentos Familiares* - POF), the evolution of the prevalence of food consumption outside the home between 2008 and 2018 had unequal behavior between the regions and urban and rural areas of Brazil, in addition to a reduction in prevalence observed in the North, South, and Southeast regions, whereas in the Northeast and Midwest regions there was an increase in the same period^7^.

Globalization and urbanization lead to the reduction of regional disparities, thus favoring the tendency of the rural population to adhere to the dietary patterns of the urban population^8^. These differences can be explained by the characteristics of the food environment, which are considerably different between urban and rural areas, considering that it ranges from eating behavior to availability in local trade and physical access to food^9^.

Brazil has a large territorial extension and differences in climate, culture, and economic activities in its regions and between its urban and rural areas, which reflect the diversity of the population’s eating habits. The 2017-2018 POF was the last one held in the country and is the most recent data source that allows us to assess differences in consumption, considering different food environments. The analysis of differences in food consumption outside the home, comparing urban and rural areas of the country, is necessary to understand elements related to the human right to adequate and healthy food and food sovereignty through the valorization and respect for food culture, which occurs differently between urban and rural areas of the country^8^.

Exploring the differences in the evolution of the participation of eating away from home between urban and rural areas of Brazil, since the early 2000s, and investigating the participation of purchased food groups in eating away from home can help in formulating intervention strategies in the eating away from home sector directed to the reality of each area. Thus, in this study we aimed to analyze the differences in the evolution of food purchase for consumption away from home between urban and rural areas of Brazil from 2002 to 2018.

## METHODS

The data used in this study come from the POF, which was carried out by the Brazilian Institute of Geography and Statistics (IBGE). For these analyses, the surveys of 2002-2003, 2008-2009, and 2017-2018 were considered. Detailed information can be found on the IBGE website (https://www.ibge.gov.br).

For the 2002-2003 POF, a two-stage cluster sampling was used. In the first stage, census tracts were selected through systematic sampling, and in the second stage, households were selected by simple random sampling without replacement. The census tracts were distributed throughout the research. The census tracts underwent geographical and statistical stratification of the sample based on the 2000 IBGE Demographic Census.

Regarding the POFs of 2008-2009 and 2017-2018, an integrated household survey system was used, which considers a master sample that facilitates the integration between the two surveys. Subsequently, a two-stage cluster sampling was carried out, the first stage being the selection of census tracts and the second, the selection of households that were distributed over the four quarters of the research. The sampling technique used in the three surveys is comparable, allowing for the selected sample to be representative of the population in the three years investigated.

The total number of households interviewed in each POF was 48,470 in the 2002-2003 period; 55,970 households in 2008-2009; and 57,920 households in 2017-2018. Only adults (aged 25 to 59 years) living in urban and rural areas of the country were considered for the study. The choice of individuals aging from 25 years onward is justified to allow their comparison according to level of education, as 25 years usually refers to the minimum time required to complete the formal educational cycle (higher education). The final sample totaled 75,304 individuals in the 2002-2003 POF; 85,587 individuals in the 2008-2009 POF; and 84,820 individuals in the 2017-2018 POF.

Data regarding the purchase of food for consumption outside the home were obtained through the individual expenditure questionnaire. Information on food expenses for consumption away from home was collected for seven consecutive days and included the purchased products, the way of obtaining them, the place of purchase, and the amount paid in the Brazilian currency (BRL). For this type of expense, the purchased amount of each food was not inquired. Eating outside the home was defined as the purchase of at least one food for consumption away from home, concerning the same consumption unit of the individual who purchased the food during the week of data collection^10^.

Foods obtained for consumption outside the home were classified into ten groups, based on the consumption and marketing characteristics of the items: alcoholic beverages, juices and refreshments, soft drinks, cereal grains, fruits, sweets, milks and derivatives, meals, fast foods, fried and baked savories. This categorization was based on the most representative characteristics of the mentioned item, and the foods consumed together were considered in more than one group (for example: savories and soft drinks were computed in the soft drinks and fried and baked savories groups)^11^.

The frequencies and their respective 95% confidence intervals (95%CI) of the purchase of food for consumption outside the home were estimated for each survey according to sociodemographic characteristics: sex, per capita family income, and level of education, according to urban and rural areas of the country. Per capita family income was stratified into four categories, based on the minimum wages in force at the time of the surveys (BRL 200.00 in 2002-2003, BRL 415.00 in 2008-2009, and BRL 954.00 in 2017-2018): up to half a minimum wage; from 0.5 to one minimum wage; from one to two minimum wages; and more than two minimum wages. Level of education was classified considering: up to four years of formal education; from four to eight years; nine to eleven years; and twelve years or over of formal education. Differences between surveys and between urban and rural areas were considered when the 95% confidence intervals of the estimates did not overlap. The frequencies of purchase of food groups for consumption outside the home were also estimated, according to urban and rural areas of Brazil and each Brazilian region, for each survey year.

Logistic regression models were developed to assess the odds of increasing or decreasing the frequency of purchasing food groups in the 2008-2009 and 2017-2018 surveys compared to the 2002-2003 survey (reference category). The models were adjusted for age and per capita family income and were separately carried out for each area (urban and rural).

The average of total expenses and of each group of foods purchased for consumption outside the home was calculated by multiplying the total amount paid by the deflator index. To compare expenditures throughout the surveys, the Extended National Consumer Price Index (*Índice de Preços ao Consumidor Amplo* - IPCA) for 2018, calculated by IBGE, was used. Subsequently, expenses for the 2002-2003 and 2008-2009 periods were updated based on the 2018 IPCA. To evaluate changes in expenses between surveys, linear regression models were used, with the year of the survey as an independent variable and the expenses of each food group as dependent variables. The models were adjusted for age and per capita family income and were separately carried out for each area (urban and rural).

The analyses were performed in the SAS (Statistical Analysis System) software, online version, considering the expansion factors and the complexity of the sample.

## RESULTS

The percentages of purchase of food for consumption outside the home in urban areas of the country increased between 2002-2003 and 2008-2009 (40.7%; 95%CI 39.8-41.5 *vs.* 47.5%; 95%CI 46.5-48.6), followed by a reduction in 2017-2018 (37.7%; 95%CI 36.8-38.5). Conversely, in the rural area, the values remained stable in the last 16 years (31.2%; 95%CI 29.7-32.7 in 2002-2003; 33.4%; 95%CI 31.6-35.1 in 2008-2009; and 30.7%; 95%CI 28.9-32.4 in 2017-2018), as shown in Table 1.

**Table 1:** Frequency (%) - 95% confidence interval - of purchasing food for away-from-home consumption by Brazilian adults, according to sociodemographic characteristics, in urban and rural areas of Brazil. Consumer Expenditure Surveys 2002-2003, 2008-2009, and 2017-2018.

Sociodemographic characteristics	Urban area			Rural area		
	2002-2003 (n=58,878)	2008-2009 (n=66,042)	2017-2018 (n=65,823)	2002-2003 (n=16,426)	2008-2009 (n=19,545)	2017-2018 (n=18,997)
	% (95%CI)	% (95%CI)	% (95%CI)	% (95%CI)	% (95%CI)	% (95%CI)
Total	40.7 (39.8-41.5)	47.5 (46.5-48.6)	37.7 (36.8-38.5)	31.2 (29.7-32.8)	33.4 (31.6-35.2)	30.7 (28.9-32.4)
Sex						
Men	46.4 (45.1-47.6)	51.9 (50.7-53.2)	39.5 (38.5-40.5)	37.2 (35.4-39.0)	36.9 (35.0-38.9)	30.5 (28.5-32.5)
Women	35.5 (34.3-36.6)	43.7 (42.4-44.9)	36.1 (35.1-37.0)	24.7 (22.7-26.8)	29.5 (27.6-31.5)	30.9 (28.8-32.9)
Level of education (years of education)						
Up to four years	28.8 (27.7-30.0)	32.7 (31.2-34.2)	22.8 (21.4-24.2)	29.4 (27.8-30.9)	29.6 (27.8-31.5)	25.5 (23.1-27.8)
Four to eight years	38.2 (36.7-39.6)	44.1 (42.6-45.7)	29.1 (27.8-30.3)	36.9 (37.7-40.1)	35.7 (33.0-38.4)	29.2 (26.9-31.4)
Nine to 11 years	48.0 (46.4-49.5)	51.9 (50.4-53.3)	31.4 (30.0-32.9)	35.2 (31.6-38.7)	41.3 (37.8-44.8)	32.8 (30.0-35.5)
Over 12 years	61.3 (59.3-63.4)	64.1 (62.3-65.9)	44.3 (43.2-45.4)	41.9 (34.9-49.0)	50.1 (44.7-55.5)	38.7 (36.2-41.1)
Income (MW*)						
<0.5 MW	25.7 (24.5-27.0)	30.4 (28.8-31.9)	23.1 (22.0-24.3)	26.2 (24.6-27.8)	27.4 (25.2-29.5)	26.7 (24.4-29.1)
0.5-1.0 MW	32.7 (31.3-34.1)	40.2 (38.7-41.7)	30.6 (29.4-31.9)	33.6 (31.3-34.1)	34.5 (32.0-37.0)	31.6 (29.2-33.9)
1-2 MW	41.4 (39.8-42.9)	48.7 (47.2-50.2)	38.1 (36.8-39.4)	36.3 (33.4-39.2)	39.8 (36.9-42.7)	34.4 (31.8-37.1)
>2 MW	54.2 (52.7-55.7)	62.3 (60.7-63.9)	52.7 (51.2-54.3)	42.4 (38.6-46.3)	48.3 (44.0-52.6)	42.0 (38.4-45.5)

MW: Minimum Wage. 95%CI: 95% confidence interval.

**Figure 1:**
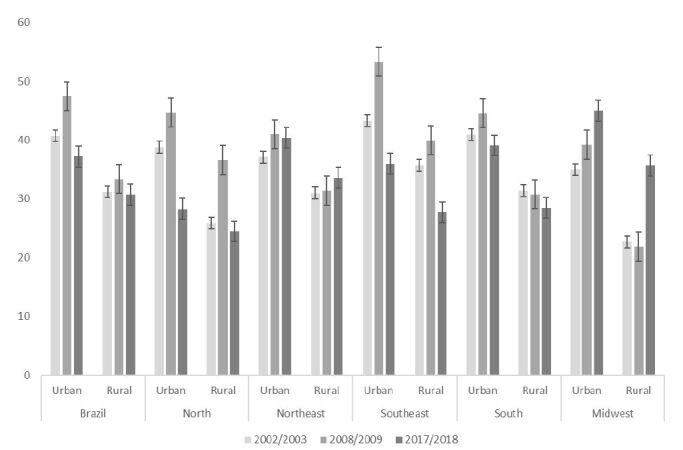
Frequency of purchasing food for away-from-home consumption by Brazilian adults, according to regions and urban and rural areas of Brazil. Consumer Expenditure Surveys 2002-2003, 2008-2009, and 2017-2018.

We found differences regarding changes in the frequency of purchasing food for consumption away from home over the years, considering urban and rural areas, depending on the Brazilian region. In the North region, we verified an increase in percentages between 2002-2003 and 2008-2009, with a subsequent reduction in 2017-2018, both for the urban and rural areas. In the Northeast region, there was an increase between 2002 and 2008, followed by stability in the period from 2008-2009 to 2017-2018 in the urban area, whereas in the rural area the participation of eating away from home has remained stable since 2002. For the Southeast region, we highlight a significant reduction in the percentages of individuals who purchased food for consumption outside the home from 2008-2009 to 2017-2018 in both areas, from 53.3 to 36.0% in the urban area and from 40.0 to 27.7% in the rural area. In the urban areas of the South region, there was a decreasing tendency for the prevalence of purchase from 2008-2009 to 2017-2018, unlike the stability observed for the entire period in the rural area. In the urban area of the Midwest, there was an increase throughout the three surveys, with the highest frequency in 2017-2018 (45%), while in the rural area there was a significant increase from 2008-2009 to 2017-2018 (21.9 vs. 35.7%), corresponding to the highest purchase frequency in this period (Figure 1).

Considering the frequencies of purchasing food for consumption outside the home, according to the food groups, there was a reduction in the percentage of purchase of soft drinks and alcoholic beverages and an increase in the frequency of purchase of meals in the 16 years investigated, both in the urban and rural areas of the country. For the urban area, we observed a reduction in the percentages for the group of juices and refreshments, sweets and fast foods. Conversely, the percentage of individuals who purchased juices and refreshments, fast foods, and fried and baked savories remained stable in the rural area between 2002 and 2018 (Table 2). The differences between urban and rural areas showed distinct behaviors, depending on the Brazilian region. Overall, there was a reduction in the frequency of purchase of soft drinks, alcoholic beverages, juices and refreshments and an increase in the purchase of meals for consumption outside the home. In the rural area of the Midwest and Northeast regions, the increase was more significant. We also observed a difference in the frequency of purchase of fast food in the rural area, which remained stable in the Northeast, Southeast, South, and Midwest regions. Conversely, there was an increase in the urban area of the Midwest; in the Northeast and South regions, frequencies remained stable; and in the North and Southeast, there was a reduction (Supplementary Material).

**Table 2:** Frequency (%) - 95% confidence interval - of purchasing food groups for away-from-home consumption by Brazilian adults, in urban and rural areas of Brazil. Consumer Expenditure Surveys 2002-2003, 2008-2009, and 2017-2018.

Food groups	Urban area			Rural area		
	2002-2003 POF (n=58,878)	2008-2009 POF (n=66,042)	2017-2018 POF (n=65,823)	2002-2003 POF (n=16,426)	2008-2009 POF (n=19,545)	2017-2018 POF (n=18,997)
	% (95%CI)	% (95%CI)	% (95%CI)	% (95%CI)	% (95%CI)	% (95%CI)
Alcoholic beverages	8.6 (8.2-9.1)	6.4 (6.0-8.0)	3.5 (3.2-3.7)	8.4 (7.7-9.2)	6.6 (5.9-7.2)	3.8 (3.3-4.2)
Juices and refreshments	5.8 (5.4-6.1)	6.0 (5.7-6.4)	3.7 (3.4-4.0)	3.1 (2.7-3.5)	3.8 (3.3-4.4)	3.9 (3.3-4.5)
Soft drinks	13.5 (12.9-14.1)	11.1 (10.5-11.7)	5.1 (4.8-5.4)	9.3 (8.5-10.1)	7.9 (7.2-8.6)	4.6 (4.0-5.2)
Cereal grains	4.2 (3.9-4.5)	4.5 (4.2-4.8)	4.9 (4.6-5.2)	4.2 (3.8-4.7)	4.2 (3.7-4.8)	5.1 (4.4-5.7)
Fruits	0.7 (0.5-0.8)	1.0 (0.9-1.1)	1.1 (1.0-1.2)	0.6 (0.4-0.8)	1.3 (1.0-1.6)	1.1 (0.9-1.4)
Sweets	9.0 (8.5-9.5)	8.3 (7.9-8.8)	5.4 (5.0-5.8)	7.4 (6.5-8.3)	5.3 (4.7-5.9)	4.0 (3.4-4.7)
Milk and dairy products	2.9 (2.6-3.2)	0.8 (0.7-0.9)	1.9 (1.7-2.0)	2.1 (1.8-2.5)	0.9 (0.6-1.2)	1.9 (1.6-2.2)
Meals	13.6 (13.0-14.3)	22.3 (21.3-23.3)	22.7 (22.0-23.4)	6.3 (5.6-7.0)	10.4 (9.4-11.3)	15.4 (14.3-16.6)
Fast foods	8.5 (8.0-9.0)	7.0 (6.6-7.5)	6.8 (6.4-7.2)	3.7 (3.2-4.1)	3.4 (2.9-3.9)	2.8 (2.4-3.2)
Fried and baked savories	9.9 (9.4-10.4)	7.3 (6.9-7.7)	8.0 (7.5-8.5)	7.6 (6.9-8.3)	6.3 (5.6-6.9)	7.1 (6.3-7.9)

95%CI: 95% confidence interval; POF: Consumer Expenditure Survey.

When analyzing the odds of purchasing food groups, we found, for the urban area, a reduction in the frequency of purchasing juices and refreshments, soft drinks, sweets, and meals; conversely, in the rural area, there was an increase for the groups of juices and refreshments and meals, and a reduction for soft drinks (Table 3).

**Table 3:** Odds ratio (adjusted for age and per capita family income) of purchasing food groups for away-from-home consumption by Brazilian adults in the 2008-2009 and 2017-2018 surveys compared to the 2002-2003 survey (reference category), according to urban and rural areas of Brazil. Consumer Expenditure Surveys 2002-2003, 2008-2009, and 2017-2018.

Food groups	Urban area			Rural area		
	2002-2003 POF	2008-2009 POF	2017-2018 POF	2002-2003 POF	2008-2009 POF	2017-2018 POF
		OR (95%CI)	OR (95%CI)		OR (95%CI)	OR (95%CI)
Alcoholic beverages	Ref.	0.72 (0.63-0.78)	0.36 (0.32-0.39)	Ref.	0.76 (0.65-0.89)	0.42 (0.35-0.50)
Juices and refreshments	Ref.	1.04 (0.94-1.14)	0.58 (0.52-0.64)	Ref.	1.26 (1.04-1.53)	1.31 (1.05-1.63)
Soft drinks	Ref.	0.80 (0.74-0.86)	0.33 (0.30-0.36)	Ref.	0.83 (0.73-0.95)	0.47 (0.39-0.55)
Cereal grains	Ref.	1.07 (0.97-1.19)	1.15 (1.04-1.28)	Ref.	0.20 (0.84-1.21)	1.23 (1.03-1.46)
Fruits	Ref.	1.56 (1.27-1.92)	1.66 (1.34-2.04)	Ref.	2.08 (1.40-3.10)	1.88 (1.23-2.87)
Sweets	Ref.	0.91 (0.84-1.00)	0.53 (0.48-0.58)	Ref.	0.70 (0.59-0.84)	0.54 (0.43-0.67)
Milk and dairy products	Ref.	0.28 (0.24-0.33)	0.63 (0.55-0.73)	Ref.	0.43 (0.30-0.61)	0.89 (0.70-1.13)
Meals	Ref.	1.67 (1.55-1.80)	1.31 (1.22-1.41)	Ref.	1.63 (1.39-1.92)	2.27 (1.94-2.66)
Fast foods	Ref.	0.80 (0.73-0.88)	0.72 (0.65-0.79)	Ref.	0.93 (0.76-1.14)	0.76 (0.61-0.94)
Fried and baked savories	Ref.	0.72 (0.66-0.78)	0.77 (0.71-0.84)	Ref.	0.83 (0.71-0.96)	0.94 (0.80-1.11)

95%CI: 95% confidence interval; POF: Consumer Expenditure Survey; OR: odds ratio.

In Table 4 we show the averages of the population’s spending on food groups for consumption outside the home. We can observe that spending has increased for almost all food groups. There was an increase in spending on the groups of meals, fast foods, and fried and baked savories for urban and rural areas of the country. When specifically analyzing each area, we observed a reduction in spending on soft drinks in urban areas and an increase in spending on alcoholic beverages, juices and refreshments in rural areas.

**Table 4:** Average spending (in BRL) - adjusted for age and per capita family income - on food groups for away-from-home consumption by Brazilian adults, according to urban and rural areas of Brazil. Consumer Expenditure Surveys 2002-2003, 2008-2009, and 2017-2018.

Food groups	Urban area			Rural area		
	2002-2003 POF	2008-2009 POF	2017-2018 POF	2002-2003 POF	2008-2009 POF	2017-2018 POF
	(95%CI)	(95%CI)	(95%CI)	(95%CI)	(95%CI)	(95%CI)
Total expenditure	402.3 (378.2-426.5)	750.8 (709.8-791.7)	1147.4* (1093.6-1201.1)	150.7 (136.4-165.0)	275.2 (246.5-304.0)	555.1* (506.8-603.3)
Alcoholic beverages	62.4 (56.8-68.0)	57.9 (52.9-63.0)	61.6** (55.5-67.7)	33.9 (29.5-38.3)	43.3 (37.7-49.0)	57.7** (44.4-70.9)
Juices and refreshments	8.9 (7.9-9.8)	15.4 (13.7-17.1)	13.5 (12.0-15.0)	2.5 2.1-2.9	5.7 (4.5-6.8)	8.6* (6.7-10.6)
Soft drinks	29.5 (27.1-31.9)	16.2 (14.3-18.2)	17.0* (15.8-18.2)	11.5 (10.2-12.8)	16.2 (14.3-18.2)	13.4 (10.9-16.0)
Cereal grains	5.6 (4.3-7.0)	7.9 (7.0-8.9)	21.6* (18.3-24.8)	3.6 (3.0-4.0)	4.6 (3.9-5.3)	12.9* (11.1-14.8)
Fruits	1.1 (0.9-1.4)	2.6 (1.9-3.4)	5.1* (4.3-5.9)	0.7 (0.4-1.0)	2.0 (1.4-2.6)	2.4** (1.6-3.2)
Sweets	13.0 (11.0-15.0)	20.5 (18.2-22.8)	33.3 (29.0-37.6)	6.8 (5.6-8.0)	7.4 (5.9-8.9)	11.6 (9.4-13.7)
Milk and dairy products	5.4 (4.6-6.2)	1.5 (1.2-1.9)	6.2 (5.3-7.1)	2.1 (1.6-2.6)	0.9 (0.6-1.2)	4.1** (3.0-5.2)
Meals	185.7 (168.0-203.4)	439.2 (407.6-470.7)	741.1* (699.7-782.6)	41.5 (35.4-47.6)	117.8 (101.6-134.1)	317.2* (283.5-350.9)
Fast foods	43.4 (39.1-47.8)	43.9 (39.5-48.4)	95.2* (87.9-102.5)	10.8 (8.4-13.1)	12.5 (9.9-15.2)	24.8** (18.7-30.9)
Fried and baked savories	16.4 (15.0-17.7)	26.4 (23.9-28.8)	46.4* (42.9-50.0)	7.5 (6.5-8.5)	13.7 (11.4-16.0)	25.1* (21.9-28.4)

95%CI: 95% confidence interval; POF: Consumer Expenditure Survey; *p<0.001 for means’ difference throughout the surveys adjusted for age and income; **p<0.05 for means’ difference throughout the surveys adjusted for age and income.

## DISCUSSION

The participation of eating away from home was higher in urban areas of all five regions of Brazil. Nevertheless, we observed a decreasing trend in the purchase percentages in the urban area over the analyzed period, whereas in the rural area this behavior remained stable in the last 16 years. However, the purchase percentages for rural areas are approaching those observed in urban areas of the country. Regarding food, there was a reduction in the purchase of soft drinks and an increase in the purchase of meals away from home.

The urbanization of the rural area has contributed to changes in lifestyle and, consequently, in dietary patterns. These changes have positive aspects regarding the greater amount of food products and a lower cost; however, it reinforces the issue of social inequality, due to the difficulty in purchasing food products with nutritional quality^12^ ^,^ ^13^.

The incorporation of urban habits in rural areas is observed in the types of purchased foods: in this study, we identified that the percentage of individuals who purchased fast foods and fried and baked savories over the years remained stable in the rural area, not following the decrease observed in the urban area. Furthermore, the frequencies of purchase for consumption outside the home of the evaluated food groups were similar in the urban and rural areas of the country in 2017-2018. This finding demonstrates the approximation of behaviors between the urban and rural areas and represents a new context of rural life. Nonetheless, they emphasize the inversion of dietary practices previously based on production for own consumption and which, nowadays, give rise to the consumption of items produced outside the home.

The reduction observed in the frequency of purchase of food for consumption outside the home, in the urban area, may be related to the increase in consumption via delivery, which was not considered as consumption outside the home by IBGE’s expenditure surveys. Thus, ready-made foods from restaurants, fast-food chains, or other establishments, if consumed at home, are classified as at-home consumption^14^. It is worth highlighting that our findings refer to the period before the new coronavirus (COVID-19) pandemic, and authors of studies carried out during this period indicate changes in eating and buying habits^15^ ^,^ ^16^, including the increasing use of delivery apps^17^. Although we observe this reality in the urban area, the increase in the frequency of purchase of some food items in the rural area or even the non-reduction in the frequency of this purchase reinforces the approximation between the two areas.

Even though authors of previous studies have associated the consumption of food outside the home with the consumption of processed foods^18^, the general scenario points to a reduction in the frequency of purchase of soft drinks and alcoholic beverages and an increase in the frequency of purchase of meals for both the urban and rural areas of Brazil. In this study, we considered meals as the items that were reported as lunch and dinner and their variations (for instance: self-service lunch meal or à la carte dinner, etc.). In Brazil, these meals are typically composed of rice, beans, and some source of protein (meat, chicken, fish, eggs, etc.)^7^, which may indicate the purchase of foods with a lower level of processing. 

The increase in the frequency of purchase of meals prepared and consumed outside the home reinforces the changes in lifestyle and, consequently, in the eating behavior of the population of the rural area of the country. It should be noted that, with urbanization, less and less time is dedicated to preparing meals, and purchasing food for consumption outside the home may be related to everyday activities, such as employment, school, or appointments, thus validating the need for quick solutions for consumption^19^.

Conversely, eating away from home is also a leisure activity^20^ and draws attention to the fact that, for the rural area, the percentages of individuals who purchased fast foods and fried and baked savories remained stable between 2002 and 2018. Thus, the difference that existed between the areas in 2002-2003 has reduced, and the frequency of purchase of food for consumption outside the home in 2017-2018 is similar between the two areas for all food groups.

These characteristics of the dietary pattern may be related to the greater availability of income, which allows affording the purchase of food for consumption outside the home^19^. In a study carried out with data from the 2008-2009 POF, in order to evaluate the evolution of expenses with eating away from home, the authors demonstrated that the improvement in income was directly associated with the expenditure on these meals^21^. According to our results, the spending increased for almost all food groups throughout the surveys; however, the frequency decreased for most of the same groups, which may indicate an increase in food prices, which mainly affects the lower income group. We used the IPCA correcting the values of 2002-2003 and 2008-2009, but even so, expenses were higher in 2017-2018, with the exception of juices in the urban area. Although our study is based on data prior to the COVID-19 pandemic, the Brazilian Association of Bars and Restaurants (*Associação Brasileira de Bares e Restaurantes* - ABRASEL) highlighted that the number of Brazilians who report spending on eating away from home is approaching pre-pandemic levels^22^.

Other factors that influence this dietary pattern are the ease of consumption and the appeal of food and beverage promotions^23^. Researchers of a previous study, with data from the 2008-2009 POF, indicated that Brazilians frequently opted for places that offered ease, convenience, and variety, such as diners and restaurants, both in rural and urban regions^24^.

Eating outside the home has been considered of worse nutritional quality when compared to eating inside the home, as it is related to higher caloric intake and smaller number of vitamins and minerals^24^ ^-^ ^26^. Researchers indicate an association between eating outside the home and higher intake of foods with saturated fat, free sugars, and higher energy density^27^. Moreover, there is evidence that the greater the frequency of consumption outside the home, the greater the intake of ultra-processed foods^24^ ^,^ ^28^, which are risk factors for the onset of NCDs and excessive weight gain^29^ ^,^ ^30^. If we consider inequities in the access to healthcare services, the changes observed in the rural area over time are worthy of attention. The demand for healthcare services is lower in the rural area, due to greater social vulnerability and greater difficulty accessing the services, as well as the lower supply of professionals in relation to the urban area^31^ ^,^ ^32^.

There are few Brazilian studies whose authors evaluated the evolution of the frequency of purchasing food for consumption outside the home focused on urban and rural areas. Our study is a pioneer in the evaluation of this evolution focusing on the rural environment of the country, and the data indicate new perspectives for residents of this area, albeit at a slower pace, but capable of currently presenting many consumption characteristics similar to those of the urban area, approximating both areas regarding eating behaviors. The adherence to the dietary habits and patterns of urban areas is increasingly common among residents of rural areas. Thus, public policies should focus on the promotion of adequate and healthy food that values the traditional food culture of Brazilians, including regulatory measures that contribute to the development of healthy food environments^8^.

As limitations of the study, we mention the lack of detail in relation to what was actually purchased and consumed. For instance: the meal group consists of items reported as à la carte lunch and dinner or self-service meals and takeout, making it impossible to detail, in these cases, the foods consumed. In addition, information on the amount of food purchased is only available for the purchase of food at home, and it is not possible to estimate the amount and calories for food purchased for consumption outside the home. Hence, the results must be interpreted with caution, considering that the decrease in purchase frequency may not reflect the decrease in the amount consumed.

Another limitation regards the classification of eating away from home used by IBGE, which considers only foods prepared and consumed outside the home, that is, foods purchased via delivery or drive-through, if consumed at home, are included in the household’s food availability. However, this classification was the same used in all surveys^7^ ^,^ ^10^.

Changes in the frequency of purchase of food for away-from-home consumption between 2002 and 2018 differed in urban and rural areas of the country. The frequency of purchase observed for the rural area is approaching that observed in urban areas, indicating a new context of rural life and the need for studies that evaluate eating behaviors in this area more thoroughly. The data highlight the importance of considering changes in the local food environment to understand the impact of purchasing food outside the home on the health of the population, thus indicating that studies on the food environment in the rural area of the country are extremely important to detect characteristics that explain the observed changes.
