# Supplementary material for: Purchase of food for away-from-home consumption according to urban and rural areas of Brazil between 2002 and 2018
Source: Rev Bras Epidemiol. 2025 Apr 7;28:e250013. doi: 10.1590/1980-549720250013 (PMC11980822; doi:10.1590/1980-549720250013)
Supplement: Translation

# Translation: Aquisição de alimentos para consumo fora do domicílio segundo áreas urbanas e rurais do Brasil entre 2002 e 2018

**DOI:** 10.1590/1980-549720250013.2

**Authors:** Thais Meirelles de Vasconcelos, Anna Karolyne Pontes de França, Marina Campos Araújo, Ilana Nogueira Bezerra

**Affiliations:** I Universidade Estadual do Ceará, Programa de Pós-Graduação em Saúde Coletiva - Fortaleza (CE), Brasil.; II Universidade Estadual do Ceará, Centro de Ciências da Saúde - Fortaleza (CE), Brasil.; III Escola Nacional de Saúde Pública Sergio Arouca, Programa de Pós-Graduação em Epidemiologia em Saúde Pública - Manguinhos (RJ), Brasil.; IV Universidade Estadual do Ceará, Programa de Pós-Graduação em Nutrição e Saúde - Fortaleza (CE), Brasil.

**Keywords:** Hábitos alimentares, Consumo alimentar, Inquéritos alimentares

## Abstract

**Objetivo:**

Analisar as diferenças na evolução da aquisição de alimentos para consumo fora do domicílio entre áreas urbanas e rurais do Brasil, no período de 2002 a 2018.

**Métodos:**

Utilizaram-se dados populacionais de 245.711 adultos (25-59 anos) das Pesquisas de Orçamentos Familiares (POF) do Instituto Brasileiro de Geografia e Estatística (IBGE) de 2002-2003, 2008-2009 e 2017-2018. Os alimentos foram agrupados em 10 categorias segundo características de consumo e de comercialização, e a frequência de aquisição de alimentos para consumo fora de casa foi comparada segundo áreas urbanas e rurais do Brasil e das grandes regiões do país. Diferenças entre as áreas foram consideradas quando os intervalos de 95% de confiança das estimativas não se sobrepuseram.

**Resultados:**

Os dados indicam um aumento na frequência de aquisição de alimentos para consumo fora de casa nas áreas urbanas entre 2002-2003 e 2008-2009, seguido de uma redução em 2017-2018. Nas áreas rurais, a frequência permaneceu estável ao longo dos anos. Com relação aos grupos de alimentos, houve redução na frequência de compra de refrigerantes e bebidas alcoólicas e um aumento na frequência de refeições fora de casa. As diferenças entre áreas urbanas e rurais variaram de acordo com as regiões do Brasil.

**Conclusão:**

A aquisição de alimentos para consumo fora de casa entre 2002 e 2018 mostrou-se diferente nas áreas urbanas e rurais do país, no entanto, a área rural está se aproximando da área urbana. Esses resultados ressaltam o novo contexto da vida rural e a necessidade de avaliar os comportamentos alimentares nessa área.

## INTRODUÇÃO

O aumento da participação da alimentação fora de casa na dieta e seus impactos negativos para a saúde são reconhecidos em países desenvolvidos e em desenvolvimento^1^ ^,^ ^2^. Algumas explicações se concentram nas mudanças demográficas e socioeconômicas que modificaram o comportamento alimentar^3^. Atrelado a isso, o consumo fora de casa está relacionado a ingestão de alimentos de alta densidade calórica, maior quantidade de gordura saturada e menor aporte de fibras^4^ ^,^ ^5^, contribuindo no desenvolvimento de Doenças Crônicas Não Transmissíveis (DCNT)^6^.

As últimas Pesquisas de Orçamentos Familiares (POF) apontaram que a evolução da prevalência do consumo alimentar fora de casa entre 2008 e 2018 teve comportamento desigual entre as regiões e as áreas urbanas e rurais do Brasil, sendo também observada a redução da prevalência nas regiões Norte, Sul e Sudeste, enquanto nas regiões Nordeste e Centro-Oeste observou-se aumento no mesmo período^7^.

A globalização e urbanização levam à redução das disparidades regionais, favorecendo, assim, a tendência de adesão da população rural aos padrões alimentares da população urbana^8^. Essas diferenças podem ser explicadas pelas características do ambiente alimentar, as quais são consideravelmente diferentes entre áreas urbanas e rurais, tendo em vista que engloba desde o comportamento alimentar até a disponibilidade no comércio local e o acesso físico aos alimentos^9^.

O Brasil possui grande extensão territorial e diferenças de clima, cultura e atividades econômicas em suas regiões e entre suas áreas urbanas e rurais, que refletem na diversidade de hábitos alimentares da população. A POF de 2017-2018 foi a última realizada no país e é a fonte de dados mais recente que permite avaliar as diferenças de consumo, considerando distintos ambientes alimentares. A análise das diferenças do consumo de alimentos fora de casa, comparando-se as áreas urbanas e rurais do país, faz-se necessária para compreender elementos relacionados ao direito humano à alimentação adequada e saudável e à soberania alimentar mediante a valorização e o respeito à cultura alimentar, que ocorre de forma diferenciada entre as áreas urbanas e rurais do país^8^.

Explorar as diferenças da evolução da participação da alimentação fora de casa entre as áreas urbana e rural do Brasil, desde o início dos anos 2000, e investigar a participação de grupos de alimentos adquiridos na alimentação fora de casa pode auxiliar na fundamentação de estratégias de intervenção no setor de alimentação fora de casa direcionadas à realidade de cada área. Dessa forma, o objetivo do presente estudo foi analisar as diferenças na evolução da aquisição de alimentos para consumo fora do domicílio entre áreas urbanas e rurais do Brasil no período de 2002 a 2018.

## MÉTODOS

Os dados utilizados no presente estudo são provenientes da POF, realizada pelo Instituto Brasileiro de Geografia e Estatística (IBGE). Para estas análises utilizaram-se as pesquisas de 2002-2003, 2008-2009 e 2017-2018. Informações detalhadas podem ser encontradas no *site* do IBGE (https://www.ibge.gov.br).

Para a POF 2002-2003 utilizou-se plano amostral por conglomerado em dois estágios. No primeiro, os setores censitários foram selecionados por meio de amostragem sistemática, e, no segundo, os domicílios foram selecionados por amostragem aleatória simples sem reposição. Foi realizada uma distribuição dos setores durante o período de duração da pesquisa. Os setores passaram por estratificação geográfica e estatística da amostra com base no Censo Demográfico de 2000 do IBGE.

Em relação às POFs de 2008-2009 e 2017-2018, utilizou-se um sistema integrado de pesquisas domiciliares que considera uma amostra mestra que facilita a integração entre as duas pesquisas. A partir disso foi realizado um plano amostral por conglomerado em dois estágios, sendo o primeiro a seleção de setores censitários e o segundo a seleção de domicílios que foram distribuídos ao longo dos quatro trimestres de duração da pesquisa. A técnica de amostragem utilizada nos três inquéritos é comparável, permitindo que a amostra selecionada seja representativa da população nos três anos investigados.

O número total de domicílios entrevistados em cada POF foi de 48.470 no período de 2002-2003, 55.970 domicílios em 2008-2009, e, no período de 2017-2018, 57.920 domicílios. Consideramos, para esse estudo, somente os adultos (25 a 59 anos) residentes nas áreas urbanas e rurais do país. A escolha por indivíduos a partir de 25 anos de idade se justifica para permitir a comparação deles de acordo com a escolaridade, uma vez que 25 anos se refere ao tempo mínimo necessário para completude do ciclo educacional (nível superior). A amostra final totalizou 75.304 indivíduos na POF 2002-2003, 85.587 indivíduos na POF 2008-2009 e 84.820 indivíduos na POF 2017-2018.

Os dados referentes à aquisição de alimentos para consumo fora de casa foram obtidos por meio do questionário de despesa individual. As informações referentes às despesas com alimentação para consumo fora do domicílio foram coletadas durante sete dias consecutivos e incluíram os produtos adquiridos, a forma de obtenção, o local de compra e o valor pago em reais. Para esse tipo de despesa, a quantidade adquirida de cada alimento não foi perguntada. Definiu-se como alimentação fora de casa a aquisição de, pelo menos, um alimento para consumo fora de casa, destinada à mesma unidade de consumo do indivíduo que adquiriu o alimento durante a semana da coleta de dados^10^.

Os alimentos obtidos para consumo fora de casa foram classificados em 10 grupos, com base nas características de consumo e de comercialização dos itens: bebidas alcoólicas, sucos e refrescos, refrigerantes, cereais, frutas, doces, leites e derivados, refeições, *fast-foods*, salgados fritos e assados. Essa categorização baseou-se nas características mais representativas do item citado, e os alimentos consumidos em conjunto foram considerados em mais de um grupo (por exemplo: salgado e refrigerante foram computados nos grupos refrigerantes e salgados fritos e assados)^11^.

Estimaram-se as frequências e seus respectivos intervalos de confiança 95% (IC95%) da aquisição de alimentos para consumo fora de casa para cada pesquisa de acordo com características sociodemográficas: sexo, renda familiar *per capita* e escolaridade, segundo áreas urbanas e rurais do país. A renda familiar *per capita* foi estratificada em quatro categorias, com base nos salários mínimos vigente à época das pesquisas (R$ 200,00 em 2002-2003, R$ 415,00 em 2008-2009 e R$ 954,00 em 2017-2018): até meio salário mínimo; de 0,5 a um salário mínimo; de um a dois salários mínimos; e mais de dois salários mínimos. A escolaridade foi classificada considerando-se: até quatro anos de estudo; de quatro a oito anos; nove a onze anos; e doze anos ou mais de estudo. Diferenças entre as pesquisas e entre as áreas urbanas e rurais foram consideradas quando os intervalos de 95% de confiança das estimativas não se sobrepuseram. Também foram estimadas as frequências de aquisição dos grupos de alimentos para consumo fora de casa, segundo áreas urbanas e rurais do Brasil e de cada região brasileira, para cada ano das pesquisas.

Modelos de regressão logística foram desenvolvidos para avaliar a chance de se aumentar ou diminuir a frequência de aquisição dos grupos de alimentos nas pesquisas de 2008-2009 e 2017-2018 em comparação ao inquérito de 2002-2003 (categoria de referência). Os modelos foram ajustados por idade e renda familiar *per capita* e conduzidos separadamente para cada área (urbana e rural).

A média dos gastos totais e de cada grupo de alimentos adquiridos para consumo fora de casa foi calculada multiplicando-se o valor total pago pelo índice deflator. Para permitir a comparação dos gastos ao longo das pesquisas, utilizou-se o Índice de Preços ao Consumidor Amplo (IPCA) do ano de 2018, calculado pelo IBGE. Em seguida, as despesas dos períodos de 2002-2003 e 2008-2009 foram atualizadas com base no IPCA de 2018. Para avaliar as mudanças nos gastos entre as pesquisas, modelos de regressão linear foram utilizados, tendo o ano do inquérito como variável independente e as despesas de cada grupo de alimentos como variáveis dependentes. Os modelos também foram ajustados por idade e renda familiar *per capita* e conduzidos separadamente para cada área (urbana e rural).

As análises foram realizadas no *software* SAS (*Statistical Analysis System*), versão *online*, considerando os fatores de expansão e a complexidade da amostra.

## RESULTADOS

Os percentuais de aquisição de alimentos para consumo fora de casa nas áreas urbanas do país aumentaram entre 2002-2003 e 2008-2009 (40,7%; IC95% 39,8-41,5 *vs.* 47.5%; IC95% 46,5-48,6), seguidos de redução em 2017-2018 (37,7%; IC95% 36.8-38.5). Em contrapartida, na área rural, mantiveram-se estáveis nos últimos 16 anos (31,2%; IC95% 29,7-32,7 em 2002-2003; 33,4%; IC95% 31,6-35,1 em 2008-2009 e 30,7%; IC95% 28,9-32,4 em 2017-2018), conforme Tabela 1.

**Tabela 1:** Frequência (%) - intervalo de confiança 95% - IC95% - de aquisição de alimentos para consumo fora de casa por adultos brasileiros, segundo características sociodemográficas, nas áreas urbanas e rurais do Brasil. Pesquisas de Orçamentos Familiares 2002-2003, 2008-2009 e 2017-2018.

Características sociodemográficas	Área urbana			Área rural		
	2002-2003 (n=58.878)	2008-2009 (n=66.042)	2017-2018 (n=65.823)	2002-2003 (n=16.426)	2008-2009 (n=19.545)	2017-2018 (n=18.997)
	% (IC95%)	% (IC95%)	% (IC95%)	% (IC95%)	% (IC95%)	% (IC95%)
Total	40,7 (39,8-41,5)	47,5 (46,5-48,6)	37,7 (36,8-38,5)	31,2 (29,7-32,8)	33,4 (31,6-35,2)	30,7 (28,9-32,4)
Sexo						
Masculino	46,4 (45,1-47,6)	51,9 (50,7-53,2)	39,5 (38,5-40,5)	37,2 (35,4-39,0)	36,9 (35,0-38,9)	30,5 (28,5-32,5)
Feminino	35,5 (34,3-36,6)	43,7 (42,4-44,9)	36,1 (35,1-37,0)	24,7 (22,7-26,8)	29,5 (27,6-31,5)	30,9 (28,8-32,9)
Escolaridade (anos de estudo)						
Até 4 anos	28,8 (27,7-30,0)	32,7 (31,2-34,2)	22,8 (21,4-24,2)	29,4 (27,8-30,9)	29,6 (27,8-31,5)	25,5 (23,1-27,8)
4 a 8 anos	38,2 (36,7-39,6)	44,1 (42,6-45,7)	29,1 (27,8-30,3)	36,9 (37,7-40,1)	35,7 (33,0-38,4)	29,2 (26,9-31,4)
9 a 11 anos	48,0 (46,4-49,5)	51,9 (50,4-53,3)	31,4 (30,0-32,9)	35,2 (31,6-38,7)	41,3 (37,8-44,8)	32,8 (30,0-35,5)
Mais de 12 anos	61,3 (59,3-63,4)	64,1 (62,3-65,9)	44,3 (43,2-45,4)	41,9 (34,9-49,0)	50,1 (44,7-55,5)	38,7 (36,2-41,1)
Renda (SM*)						
<0,5 SM	25,7 (24,5-27,0)	30,4 (28,8-31,9)	23,1 (22,0-24,3)	26,2 (24,6-27,8)	27,4 (25,2-29,5)	26,7 (24,4-29,1)
0,5-1,0 SM	32,7 (31,3-34,1)	40,2 (38,7-41,7)	30,6 (29,4-31,9)	33,6 (31,3-34,1)	34,5 (32,0-37,0)	31,6 (29,2-33,9)
1-2 SM	41,4 (39,8-42,9)	48,7 (47,2-50,2)	38,1 (36,8-39,4)	36,3 (33,4-39,2)	39,8 (36,9-42,7)	34,4 (31,8-37,1)
>2 SM	54,2 (52,7-55,7)	62,3 (60,7-63,9)	52,7 (51,2-54,3)	42,4 (38,6-46,3)	48,3 (44,0-52,6)	42,0 (38,4-45,5)

*SM: salário mínimo.

As mudanças na frequência de aquisição de alimentos para consumo fora de domicílio ao longo dos anos, considerando as áreas urbanas e rurais, apresentaram diferenças, a depender da região brasileira. A região Norte apresentou aumento nos percentuais entre 2002-2003 e 2008-2009, com posterior redução em 2017-2018, tanto para a área urbana quanto para a rural. Na região Nordeste, observa-se um aumento entre 2002 e 2008, seguido de estabilidade no período de 2008-2009 a 2017-2018 na área urbana, enquanto, na área rural, a participação da alimentação fora de casa se manteve constante desde 2002. Para a região Sudeste, destaca-se redução importante dos percentuais de indivíduos que adquiriram alimentos para consumo fora de casa de 2008-2009 para 2017-2018, em ambas as áreas, passando de 53,3 para 36,0% na área urbana e de 40,0 para 27,7% na área rural. Nas áreas urbanas da região Sul, verificou-se uma tendência de redução da prevalência da aquisição de 2008-2009 a 2017-2018, diferente da estabilidade observada para todo o período na área rural. A área urbana do Centro-Oeste apresentou aumento ao longo das três pesquisas, com destaque para a maior frequência em 2017-2018 (45%), enquanto, na área rural, houve aumento expressivo de 2008-2009 para 2017-2018 (21,9 vs. 35,7%), correspondendo à maior frequência de aquisição desse período (Figura 1).

**Figura 1:** Frequência de aquisição de alimentos para consumo fora de casa por adultos brasileiros, segundo regiões e áreas urbanas e rurais do Brasil. Pesquisas de Orçamentos Familiares 2002-2003, 2008-2009 e 2017-2018.

As frequências de aquisição de alimentos para consumo fora de casa segundo os grupos de alimentos sinalizaram redução do percentual de aquisição de refrigerantes e bebidas alcoólicas e aumento na frequência de aquisição das refeições nos 16 anos investigados, tanto na área urbana quanto na área rural do país. Para a área urbana, observou-se redução dos percentuais para o grupo dos sucos e refrescos, doces e *fast-foods*. Por outro lado, o percentual de indivíduos que adquiriram sucos e refrescos, *fast-foods* e salgados fritos e assados permaneceu constante na área rural entre 2002 e 2018 (Tabela 2). As diferenças entre as áreas urbanas e rurais mostraram comportamentos distintos, a depender da região brasileira. De uma maneira geral, houve redução na frequência de aquisição de refrigerante, bebida alcoólica, sucos e refrescos e aumento na aquisição de refeições para consumo fora de casa. Na área rural das regiões Centro-Oeste e Nordeste o aumento foi mais expressivo. Outra diferença foi na frequência de aquisição de *fast-food* na área rural, que se manteve estável nas regiões Nordeste, Sudeste, Sul e Centro-Oeste, enquanto, na área urbana do Centro-Oeste, houve aumento, no Nordeste e Sul, manutenção das frequências, e, no Norte e Sudeste, redução (Material Suplementar).

**Tabela 2:** Frequência (%) - IC95% - de aquisição de grupos de alimentos para consumo fora de casa por adultos brasileiros, nas áreas urbanas e rurais do Brasil. POF 2002-2003, 2008-2009, 2017-2018.

Grupos de alimentos	Urbano			Rural		
	POF 2002-2003 (n=58.878)	POF 2008-2009 (n=66.042)	POF 2017-2018 (n=65.823)	POF 2002-2003 (n=16.426)	POF 2008-2009 (n=19.545)	POF 2017-2018 (n=18.997)
	% (IC95%)	% (IC95%)	% (IC95%)	% (IC95%)	% (IC95%)	% (IC95%)
Bebidas alcoólicas	8,6 (8,2-9,1)	6,4 (6,0-6,8)	3,5 (3,2-3,7)	8,4 (7,7-9,2)	6,6 (5,9-7,2)	3,8 (3,3-4,2)
Sucos e refrescos	5,8 (5,4-6,1)	6,0 (5,7-6,4)	3,7 (3,4-4,0)	3,1 (2,7-3,5)	3,8 (3,3-4,4)	3,9 (3,3-4,5)
Refrigerantes	13,5 (12,9-14,1)	11,1 (10,5-11,7)	5,1 (4,8-5,4)	9.3 (8,5-10,1)	7.9 (7,2-8,6)	4,6 (4,0-5,2)
Cereais	4,2 (3,9-4,5)	4,5 (4,2-4,8)	4,9 (4,6-5,2)	4,2 (3,8-4,7)	4,2 (3,7-4,8)	5,1 (4,4-5,7)
Frutas	0,7 (0,5-0,8)	1,0 (0,9-1,1)	1,1 (1,0-1,2)	0,6 (0,4-0,8)	1,3 (1,0-1,6)	1,1 (0,9-1,4)
Doces	9,0 (8,5-9,5)	8,3 (7,9-8,8)	5,4 (5,0-5,8)	7,4 (6,5-8,3)	5,3 (4,7-5,9)	4,0 (3,4-4,7)
Leites e derivados	2,9 (2,6-3,2)	0,8 (0,7-0,9)	1,9 (1,7-2,1)	2,1 (1,8-2,5)	0,9 (0,6-1,2)	1,9 (1,6-2,2)
Refeições	13,6 (13,0-14,3)	22,3 (21,3-23,3)	22,7 (22,0-23,4)	6,3 (5,6-7,0)	10,4 (9,4-11,3)	15,4 (14,3-16,6)
*Fast-foods*	8,5 (8,0-9,0)	7,0 (6,6-7,5)	6,8 (6,4-7,2)	3,7 (3,2-4,1)	3,4 (2,9-3,9)	2,8 (2,4-3,2)
Salgados fritos e assados	9,9 (9,4-10,4)	7,3 (6,9-7,7)	8,0 (7,5-8,5)	7,6 (6,9-8,3)	6,3 (5,6-6,9)	7,1 (6,3-7,9)

IC95%: intervalo de confiança 95%; POF: Pesquisas de Orçamentos Familiares.

Ao analisarmos as chances de compra dos grupos de alimentos, verificamos, para a área urbana, redução da frequência de aquisição para sucos e refrescos, refrigerantes, doces e refeições, enquanto, na área rural, houve aumento para os grupos dos sucos e refrescos e para as refeições e redução para os refrigerantes (Tabela 3).

**Tabela 3:** Razão de chances (ajustadas por idade e renda familiar *per capita*) de adquirir grupos de alimentos para consumo fora de casa por adultos brasileiros nas pesquisas de 2008-2009 e 2017-2018 em comparação ao inquérito de 2002-2003 (categoria de referência), segundo áreas urbanas e rurais do Brasil. POF 2002-2003, 2008-2009 e 2017-2018.

Grupos de alimentos	Urbano			Rural		
	POF 2002-2003	POF 2008-2009	POF 2017-2018	POF 2002-2003	POF 2008-2009	POF 2017-2018
		RC (IC95%)	RC (IC95%)		RC (IC95%)	RC (IC95%)
Bebidas alcoólicas	Ref.	0,72 (0,63-0,78)	0,36 (0,32-0,39)	Ref.	0,76 (0,65-0,89)	0,42 (0,35-0,50)
Sucos e refrescos	Ref.	1,04 (0,94-1,14)	0,58 (0,52-0,64)	Ref.	1,26 (1,04-1,53)	1,31 (1,05-1,63)
Refrigerantes	Ref.	0,80 (0,74-0,86)	0,33 (0,30-0,36)	Ref.	0.83 (0.73-0,95)	0.47 (0,39-0,55)
Cereais	Ref.	1,07 (0,97-1,19)	1,15 (1,04-1,28)	Ref.	1,00 (0,84-1,21)	1,23 (1,03-1,46)
Frutas	Ref.	1,56 (1,27-1,92)	1,66 (1,34-2,04)	Ref.	2,08 (1,40-3,10)	1,88 (1,23-2,87)
Doces	Ref.	0,91 (0,84-1,00)	0,53 (0,48-0,58)	Ref.	0,70 (0,59-0,84)	0,54 (0,43-0,67)
Leites e derivados	Ref.	0,28 (0,24-0,33)	0,63 (0,55-0,73)	Ref.	0,43 (0,30-0,61)	0,89 (0,70-1,13)
Refeições	Ref.	1,67 (1,55-1,80)	1,31 (1,22-1,41)	Ref.	1,63 (1,39-1,92)	2,27 (1,94-2,66)
*Fast-foods*	Ref.	0,80 (0,73-0,88)	0,72 (0,65-0,79)	Ref.	0,93 (0,76-1,14)	0,76 (0,61-0,94)
Salgados fritos e assados	Ref.	0,72 (0,66-0,78)	0,77 (0,71-0,84)	Ref.	0,83 (0,71-0,96)	0,94 (0,80-1,11)

IC95%: intervalo de confiança 95%; POF: Pesquisas de Orçamentos Familiares; RC: razão de chances.

Na Tabela 4 são apresentadas as médias dos gastos da população com os grupos de alimentos para consumo fora de casa. Podemos observar que os gastos aumentaram para quase todos os grupos de alimentos. Verificou-se aumento dos gastos com os grupos das refeições, *fast-foods* e salgados fritos e assados para as áreas urbanas e rurais do país. Olhando especificamente para cada área, observamos redução dos gastos com refrigerantes nas áreas urbanas e aumento dos gastos com bebidas alcoólicas, sucos e refrescos nas áreas rurais.

**Tabela 4:** Média de gastos (em reais) - ajustada por idade e renda familiar *per capita* - com grupos de alimentos para consumo fora de casa por adultos brasileiros, segundo áreas urbanas e rurais do Brasil. POF 2002-2003, 2008-2009 e 2017-2018.

Grupos de alimentos	Urbano			Rural		
	POF 2002-2003	POF 2008-2009	POF 2017-2018	POF 2002-2003	POF 2008-2009	POF 2017-2018
	(IC95%)	(IC95%)	(IC95%)	(IC95%)	(IC95%)	(IC95%)
Gasto total	402,3 (378,2-426,5)	750,8 (709,8-791,7)	1147,4* (1093,6-1201,1)	150,7 (136,4-165,0)	275,2 (246,5-304,0)	555,1* (506,8-603,3)
Bebidas alcoólicas	62,4 (56,8-68,0)	57,9 (52,9-63,0)	61,6** (55,5-67,7)	33,9 (29,5-38,3)	43,3 (37,7-49,0)	57,7** (44,4-70,9)
Sucos e refrescos	8,9 (7,9-9,8)	15,4 (13,7-17,1)	13,5 (12,0-15,0)	2,5 (2,1-2,9)	5,7 (4,5-6,8)	8,6* (6,7-10,6)
Refrigerantes	29,5 (27,1-31,9)	16,2 (14,3-18,2)	17,0* (15,8-18,2)	11,5 (10,2-12,8)	16,2 (14,3-18,2)	13,4 (10,9-16,0)
Cereais	5,6 (4,3-7,0)	7,9 (7,0-8,9)	21,6* (18,3-24,8)	3,6 (3,0-4,0)	4,6 (3,9-5,3)	12,9* (11,1-14,8)
Frutas	1,1 (0,9-1,4)	2,6 (1,9-3,4)	5,1* (4,3-5,9)	0,7 (0,4-1,0)	2,0 (1,4-2,6)	2,4** (1,6-3,2)
Doces	13,0 (11,0-15,0)	20,5 (18,2-22,8)	33,3 (29,0-37,6)	6,8 (5,6-8,0)	7,4 (5,9-8,9)	11,6 (9,4-13,7)
Leites e derivados	5,4 (4,6-6,2)	1,5 (1,2-1,9)	6,2 (5,3-7,1)	2,1 (1,6-2,6)	0,9 (0,6-1,2)	4,1** (3,0-5,2)
Refeições	185,7 (168,0-203,4)	439,2 (407,6-470,7)	741,1* (699,7-782,6)	41,5 (35,4-47,6)	117,8 (101,6-134,1)	317,2* (283,5-350,9)
*Fast-foods*	43,4 (39,1-47,8)	43,9 (39,5-48,4)	95,2* (87,9-102,5)	10,8 (8,4-13,1)	12,5 (9,9-15,2)	24,8** (18,7-30,9)
Salgados fritos e assados	16,4 (15,0-17,7)	26,4 (23,9-28,8)	46,4* (42,9-50,0)	7,5 (6,5-8,5)	13,7 (11,4-16,0)	25,1* (21,9-28,4)

IC95%: intervalo de confiança 95%; POF: Pesquisas de Orçamentos Familiares; *p<0,001 para diferença das médias ao longo das pesquisas ajustada por idade e renda; **p<0,05 para diferença das médias ao longo das pesquisas ajustada por idade e renda.

## DISCUSSÃO

A participação da alimentação fora de casa foi superior nas áreas urbanas de todas as cinco regiões do Brasil, no entanto, observa-se tendência de redução nos percentuais de aquisição na área urbana ao longo do período analisado, enquanto, na área rural, esse comportamento permaneceu constante nos últimos 16 anos. Todavia, os percentuais de aquisição para as áreas rurais estão se aproximando dos observados nas áreas urbanas do país. Em relação aos alimentos, houve redução na frequência de compra de refrigerantes e aumento na frequência de compra de refeições fora de casa.

A urbanização da área rural tem contribuído para a mudança do estilo de vida e, consequentemente, do padrão alimentar. Essas transformações apresentam aspectos positivos, relacionados à maior quantidade de alimentos produzidos e um menor custo, porém, reforça a questão da desigualdade social, pela dificuldade de aquisição de produtos alimentícios com qualidade nutricional^12^ ^,^ ^13^.

A incorporação de hábitos urbanos no meio rural é observada nos tipos de alimentos adquiridos; nosso estudo identificou que o percentual de indivíduos que adquiriram *fast-foods* e salgados fritos e assados ao longo dos anos permaneceu constante na área rural, não acompanhando a redução observada na área urbana. Além disso, as frequências de aquisição para consumo fora de casa dos grupos de alimentos avaliados foram semelhantes na área urbana e rural do país em 2017-2018. Esse achado demonstra a aproximação de comportamentos entre a área urbana e rural e representa um novo contexto da vida rural, porém, reforçam a inversão de práticas alimentares antes baseadas na produção para consumo próprio e que hoje dão lugar ao consumo de itens produzidos fora de casa.

A redução observada na frequência de aquisição de alimentos para consumo fora de casa, na área urbana, pode estar relacionada ao aumento do consumo via *delivery*, que não foi considerado consumo fora de casa pelas pesquisas orçamentárias do IBGE. Assim, os alimentos prontos para consumo provenientes de restaurantes, *fast-foods* ou outros estabelecimentos, se consumidos em casa, são classificados como consumo dentro de casa^14^. É importante destacar que nossos achados se referem ao período anterior à pandemia de COVID-19, e estudos realizados nesse período sinalizam mudanças nos hábitos alimentares e de compras^15^ ^,^ ^16^, incluindo o crescente uso de aplicativos de entrega para compra^17^. Apesar de observarmos essa realidade na área urbana, o aumento na frequência de aquisição de alguns itens alimentares na área rural ou mesmo a não redução na frequência dessa aquisição reforça a aproximação entre as duas áreas.

Apesar de trabalhos prévios associarem consumo de alimentos fora de casa a consumo de itens processados^18^, o cenário geral aponta para a redução da frequência de aquisição de refrigerantes e bebidas alcoólicas e para o aumento na frequência de compra das refeições tanto para a área urbana quanto para a área rural do Brasil. Neste estudo considerou-se como refeições os itens que foram relatados como almoço e jantar e suas variações (por exemplo: almoço em *self*-*service* ou jantar *à la carte* etc.). No Brasil, essas refeições são tipicamente compostas por arroz, feijão e alguma fonte de proteína (carne, frango, peixe, ovos etc.)^7^, podendo indicar a aquisição de alimentos com menor nível de processamento. 

O aumento na frequência de aquisição das refeições preparadas e consumidas fora de casa é um aspecto que reforça as mudanças no estilo de vida e, consequentemente, no comportamento alimentar da população da área rural do país. Salienta-se que, com a urbanização, cada vez menos tempo é dedicado à preparação de refeições, e adquirir alimentos para consumo fora de casa pode estar relacionado a atividades da rotina, como emprego, escola ou compromissos, validando, assim, a necessidade de soluções rápidas para consumo^19^.

Por outro lado, a alimentação fora de casa também se configura como uma atividade de lazer^20^ e chama atenção o fato de que, para a área rural, os percentuais de indivíduos que adquiriram *fast-foods* e salgados fritos e assados permaneceram constantes entre 2002 e 2018. Assim, a diferença que existia entre as áreas em 2002-2003 reduziu, e a frequência de aquisição dos alimentos para consumo fora de casa em 2017-2018 está semelhante entre as duas áreas para todos os grupos de alimentos.

Essas características do padrão alimentar podem estar relacionadas à maior disponibilidade de renda, a qual permite a possibilidade de custear aquisição de alimentos para consumo fora do lar^19^. Em estudo realizado com dados da POF 2008-2009, a fim de se avaliar a evolução das despesas com alimentação fora de casa, demonstrou-se que a melhora da renda esteve diretamente associada com o dispêndio com essas refeições^21^. Nossos resultados sinalizam que o gasto aumentou para quase todos os grupos de alimentos ao longo das pesquisas, no entanto, a frequência diminui para a maioria dos mesmos grupos, o que pode indicar aumento no preço dos alimentos, que afeta principalmente a faixa de menor renda. Utilizamos o IPCA corrigindo os valores de 2002-2002 e 2008-2009, mas, ainda assim, os gastos foram maiores em 2017-2018, com exceção dos sucos na área urbana. Apesar de nosso estudo ser baseado em dados anteriores à pandemia da COVID-19, Apesar de nosso estudo ser baseado em dados anteriores à pandemia da COVID-19, a Associação Brasileira de Bares e Restaurantes (ABRASEL) destacou que o número de brasileiros que relatam gastos com alimentação fora de casa está se aproximando dos níveis pré-pandemia^22^.

Outros fatores que influenciam esse padrão alimentar são a facilidade de consumo e o apelo das promoções de alimentos e bebidas^23^. Estudo realizado anteriormente, com dados da POF 2008-2009, sinalizou que os brasileiros optaram frequentemente por locais que ofereciam facilidade, comodidade e variedade, como as lanchonetes e os restaurantes, tanto nas regiões rurais como nas urbanas^24^.

A alimentação fora de casa tem sido considerada de pior qualidade nutricional, quando comparada à alimentação dentro do lar, pois está relacionada a maior ingestão calórica e menor quantidade de vitaminas e minerais^24^ ^-^ ^26^. Estudos sinalizam associação entre comer fora de casa e maior ingestão de gordura saturada, açúcares livres e maior densidade energética^27^. Além disso, existem evidências de que quanto maior a frequência de consumo fora de casa, maior a ingestão de alimentos ultraprocessados^24^ ^,^ ^28^, os quais são fatores de risco para o aparecimento de DCNT e ganho de peso excessivo^29^ ^,^ ^30^. Se considerarmos as iniquidades no acesso aos serviços de saúde, as mudanças observadas na área rural ao longo do tempo merecem um olhar diferenciado. A procura por serviços de saúde é menor na área rural, devido à maior vulnerabilidade social e à maior dificuldade de acesso aos serviços, assim como à menor oferta de profissionais, em relação à área urbana^31^ ^,^ ^32^.

São escassos os estudos brasileiros que avaliaram a evolução da frequência de aquisição de alimentos para consumo fora de casa com foco nas áreas urbanas e rurais. Nosso estudo é pioneiro na avaliação da evolução com foco no meio rural do país, e os dados sinalizam novas perspectivas para moradores dessa área, ainda que em uma velocidade menor, porém capaz de apresentar atualmente muitas características de consumo similares aos da área urbana, aproximando ambas as áreas quanto aos comportamentos alimentares. Acredita-se que a adesão aos hábitos e padrões alimentares das áreas urbanas seja cada vez mais comum entre os residentes das áreas rurais. Dessa forma, o foco das políticas públicas deve ser a promoção da alimentação adequada e saudável que valorize a cultura alimentar tradicional dos brasileiros, incluindo medidas regulatórias que contribuam para a construção de ambientes alimentares saudáveis^8^.

Como limitações do estudo podemos citar a ausência de detalhamento em relação ao que foi realmente adquirido e consumido. Por exemplo: o grupo das refeições é composto por itens relatados como almoço e jantar *à la carte* ou *self-service* e quentinhas, sendo impossível detalhar, nesses casos, os alimentos consumidos. Além disso, informações quanto à quantidade de alimento adquirida só estão disponíveis para aquisição de alimentos no domicílio, não sendo possível estimar quantidade e calorias para alimentos adquiridos para consumo fora de casa. Assim, os resultados precisam ser interpretados com cautela, uma vez que a redução na frequência de aquisição pode não refletir a redução na quantidade consumida.

Outra limitação refere-se à classificação da alimentação fora de casa utilizada pelo IBGE, a qual considera apenas alimentos preparados e consumidos fora de casa, ou seja, alimentos adquiridos por *delivery* ou *drive-thru*, se consumidos em casa, estão incluídos na disponibilidade alimentar do agregado familiar. No entanto, esta classificação foi a mesma utilizada em todas as pesquisas^7^ ^,^ ^10^.

As mudanças na frequência de aquisição de alimentos para consumo fora de casa entre 2002 e 2018 mostraram-se diferentes nas áreas urbanas e rurais do país. A frequência de aquisição observada para a área rural está se aproximando à observada nas áreas urbanas, indicando um novo contexto da vida rural e a necessidade de estudos que avaliem de forma mais detalhada comportamentos alimentares nessa área. Os dados destacam a importância de considerar as mudanças no ambiente alimentar local para entender o impacto da aquisição de alimentos fora de casa na saúde da população, sinalizando, assim, que estudos que avaliem o ambiente alimentar na área rural do país são de extrema importância para detectar características que explicam as mudanças observadas.
